# Arthropod-Borne Virus Surveillance as a Tool to Study the Australian Mosquito Virome

**DOI:** 10.3390/v14091882

**Published:** 2022-08-26

**Authors:** Agathe M. G. Colmant, David Warrilow, Sonja Hall-Mendelin, Michael Onn, Jody Hobson-Peters, Bixing Huang, Nina Kurucz, Allan Warchot, Bridgette R. Primmer, Sally Isberg, Helle Bielefeldt-Ohmann, Roy A. Hall

**Affiliations:** 1 Unité des Virus Émergents (UVE: Aix-Marseille Univ-IRD 190-Inserm 1207), 13005 Marseille, France; 2 Australian Infectious Diseases Research Centre, School of Chemistry and Molecular Biosciences, The University of Queensland, St. Lucia 4072, Australia; 3 Public Health Virology Laboratory, Queensland Health Forensic and Scientific Services, P.O. Box 594, Archerfield 4108, Australia; 4 Brisbane City Council, Field Services, Brisbane 4000, Australia; 5 Medical Entomology, Centre for Disease Control, Public Health Unit, Top End Health Service, Darwin 0810, Australia; 6 Centre for Crocodile Research, P.O. Box 329, Noonamah 0837, Australia

**Keywords:** mosquito virus screening, macula-like virus, insect-specific flavivirus, MAVRIC, mosquito virus, insect virus

## Abstract

Mosquitoes (*n* = 4381 in 198 pools) were collected in March and April 2018 to survey the presence of West Nile virus Kunjin strain in mosquito populations around crocodile farms in the Darwin region of the Northern Territory (NT) of Australia. While no Kunjin virus was detected in these mosquitoes, we applied our viral replicative intermediates screening system termed monoclonal antibodies to viral RNA intermediates in cells or MAVRIC to this set of samples. This resulted in the detection of 28 pools with virus replicating in C6/36 mosquito cells and the identification of three insect viruses from three distinct virus classes. We demonstrate the persistence of the insect-specific flavivirus Palm Creek virus in *Coquillettidia xanthogaster* mosquitoes from Darwin over almost a decade, with limited genetic drift. We also detected a novel Hubei macula-like virus 3 strain in samples from two mosquito genera, suggesting the virus, for which the sequence was originally detected in spiders and soybean thrips, might be involved in a horizontal transmission cycle between arthropods and plants. Overall, these data demonstrate the strength of the optimized MAVRIC system and contribute to our general knowledge of the mosquito virome and insect viruses.

## 1. Main Text

Surveillance of the prevalence of arthropod-borne viruses in mosquitoes is key to the control of arthropod-borne diseases in humans and animals in Australia. There are over a hundred mosquito species in the Northern Territory (NT), which include all the major potential and actual mosquito-borne disease vectors in Australia, except for *Aedes aegypti* (Linnaeus, 1762), which is only found in Queensland [1]. The most abundant mosquito species detected in the NT are *Culex annulirostris* (Skuse, 1889), *Ae. vigilax* (Skuse, 1889) and *Coquilletidia xanthogaster* (Edwards, 1924) [2]. Medically significant mosquito-borne viruses present in the NT include the flaviviruses Murray Valley encephalitis virus and West Nile virus Kunjin strain (WNV_KUN_), as well as the alphaviruses Ross River virus, Barmah Forest virus and Sindbis virus [1,2].

In this context, mosquitoes were collected, using encephalitis virus surveillance (EVS) traps baited with CO_2_, around two crocodile farms in the Darwin region of the NT. The aim of this surveillance was initially to monitor the WNV_KUN_ distribution in mosquitoes of this region as it had been detected in farmed crocodiles of the area [3,4,5].

Between three and six overnight traps were set at various locations within two crocodile farms in rural areas of the Darwin region in the NT. Traps were set in the afternoon and collected the following morning after sunrise, fortnightly between the 15 March and the 27 April 2018. This corresponds to the middle of the peak arbovirus activity period (between January and May) and of the WNV_KUN_ high risk period (January to July) [2,5]. Twenty-three traps were analyzed in total. Overall, 4381 mosquitoes were collected, identified and sorted into 198 pools. Two mosquitoes were identified as males (one *Cx. annulirostris* and one *Cx. quinquefasciatus* (Say, 1823)) and the 4879 remaining mosquitoes were identified as females. The majority of the mosquitoes collected were *Cx. annulirostris* (62%) followed by *Mansonia uniformis* (12%) (Theobald, 1901), *Anopheles bancroftii* (7%) (Giles, 1902), *Cx. palpalis* (6%) (Taylor, 1912), *Cx. pullus* (4%) (Theobald, 1905), *Cq. xanthogaster* (2%), *Cx. quinquefasciatus* (2%) and 14 other species; see Table 1.

To maintain the required cold chain for virus isolation work, mosquito traps were transported from site in an insulated container with dry ice before mosquitoes were stored at −80 °C. Mosquito identification to species level was performed on cold tables at the Medical Entomology laboratory in Darwin using taxonomic keys. Identified mosquito species were stored in vials up to 50 specimens. Mosquito pools were shipped to the laboratory on dry ice and then stored at −80 °C until processing for virus isolation. The mosquito pools were homogenized in 2 mL tubes in Roswell Park Memorial Institute (RPMI) medium with 2% fetal bovine serum (FBS), 50 U penicillin/mL, 50 μg streptomycin/mL and 2 mM l-glutamine, with two glass beads following a cycle of 3 min at 30 Hz in a Tissue Lyser III (Qiagen, Hilden, Germany). The homogenates were clarified by centrifugation at 12,000× *g* for 10 min then filtered through a 0.8/0.2 µm sterile filter. The filtered homogenates were inoculated in four wells of a 96-well plate pre-seeded with C6/36 mosquito cells in 5% FBS RPMI with the same additives (penicillin, streptomycin and glutamine) as above and left to incubate for five days at 28 °C. These cells were selected to maximize the chances of detecting all mosquito-borne viruses present in the samples. After incubation, the supernatants were harvested and passaged in a similar fashion for three passages and the leftover supernatants stored at −80 °C. The inoculated cells were fixed in either 20% acetone in phosphate buffered saline (PBS; 140 mM NaCl, 2.7 mM KCl, 6 mM Na_2_HPO_4_, 0.9 mM KH_2_PO_4_) with 0.02% bovine serum albumin for two hours at 4 °C or 4% formaldehyde in PBS with 0.5% TritonX-100 for ten minutes at 4 °C. We performed a fixed-cell ELISA as described previously using pan-flavivirus monoclonal antibodies (mAbs) 4G2 [6] and 4G4 [7] on the acetone-fixed first passage plates and using anti-double-stranded RNA mAb 3G1 (monoclonal antibodies to viral RNA intermediates in cells—MAVRIC) on the formaldehyde-fixed third passage plates [8,9]. None of the samples were reactive in the flavivirus-specific ELISA. C6/36 cells are not the most sensitive cell line to WNV_KUN_ replication, so using vertebrate cells instead may have led to some WNV_KUN_ detections. However, this experimental design enabled the identification of 28 pools (14.14%) with replicating virus, positive for dsRNA in the MAVRIC ELISA after three passages on C6/36 cells.

These 28 samples were selected for further identification, first using a panel of insect-specific virus mAbs, developed in our laboratory, to screen for viruses known to circulate in Australian mosquito populations. We found that none of the samples contained the seadornavirus Liao ning virus (mAb 6E6) [10], negeviruses Ngewotan virus, Bustos virus or Castlerea virus (mAbs N.5C11, B.2H10 or anti-Castlerea virus mouse serum, respectively) [11,12], *Aedes* birnavirus (mAb A7E6) [13] or *Alphamesonivirus-4* Casuarina virus (mAb C.5D3) [14]. However, we were able to identify *Alphamesonivirus-1* Nam Dinh virus (mAb N.4H7) in three *Culex* pools, with a prevalence of 1.52% (3/198 pools), see Table 2 [14]. These isolates and the negative birnavirus results have already been included in other publications [13,14] but are mentioned here in an effort to show comprehensive results for this surveillance study.

In total, 10 of the remaining 25 unidentified MAVRIC-positive samples had inconsistent MAVRIC-binding profiles depending on the fixative used, and were suspected to be insect-specific flaviviruses, undetected by flavivirus mAbs 4G2 and 4G4; see O’Brien et al., 2021 for details on this peculiar binding profile [9]. We therefore proceeded to extract RNA from inoculated cell culture supernatant from the first or second passage using the Nucleospin RNA Virus isolation kit (Macherey Nagel, Düren, Germany), following the manufacturer’s protocol. These extracts were used in a reverse transcription polymerase chain reaction (RT-PCR; SuperScript III One-Step RT-PCR System with Platinum Taq DNA Polymerase (Invitrogen, Thermo Fisher Scientific, Waltham, MA, USA)) using pan-flavivirus primers FU2 and cFD3 (melting temperature 45 °C and 50 s extension time) [15]. Six samples were positive, all from *Cq. xanthogaster* mosquitoes. The RT-PCR products were gel purified using the Nucleospin gel and PCR clean up kit (Macherey Nagel, Düren, Germany) following the manufacturer’s instructions and subjected to sequencing by the Sanger method at the Australian Genome Research Facility (Brisbane, Australia).

All sequenced samples were identified as Palm Creek virus (PCV) (see Table 2, Genbank accession numbers MW959131 to MW959136) an insect-specific flavivirus first isolated from a pool of *Cq. xanthogaster* mosquitoes collected in Darwin in 2010 [16], and subsequently isolated from two pools of *Cq. xanthogaster* mosquitoes collected from Kununurra, Western Australia, in 2010 [17]. The prevalence of PCV in our samples was 3.03% (6 positives out of 198 pools), and more specifically, 37.5% (6/16 pools) in *Cq. xanthogaster*, the only species in which PCV has been detected so far. The calculated minimum infection rate in this mosquito species in our samples was 6.82% (6/88 *Cq. xanthogaster*).

A MUSCLE alignment was performed on the 737 nucleotide and corresponding 252 amino acid sequences to compare the new isolates to each other and to the two published reference PCV genomes (Genbank accession numbers KC505248.1 and KT192550.1) using Geneious Prime (Biomatters, Auckland, New Zealand). The calculated percentage identities for nucleotide and amino acid are displayed in Table 3. Despite eight years separating the isolation of the prototype strains and the new isolates, the sequences were very similar (>95% nucleotide identity; >99% amino acid identity), though not identical, in the highly conserved NS5 region sequenced.

We selected one sample from the remaining 19 unidentified MAVRIC-positive pools, DS16 (a pool of 8 *Cx. annulirostris*) and extracted RNA from the third passage supernatant using the Nucleospin RNA Virus isolation kit (Macherey Nagel, Düren, Germany), following the manufacturer’s protocol—without carrier RNA. This extract was then deep sequenced as follows. RNA was treated with Heat & Run DNase (ArcticZymes, Tromsø, Norway) to remove contaminating host DNA, and the DNAse was heat inactivated at 80 °C for five minutes. First strand cDNA was generated using Protoscript II (New England Biolabs, Ipswich, MA, USA), using the supplied random primer mix and reaction conditions as recommended by the manufacturer. The reaction product was then converted to double-stranded DNA (16 °C for 60 min) using an enzyme mixture consisting of *E. coli* DNA ligase, DNA polymerase I and RNase H (New England Biolabs, Ipswich, MA, USA), followed by heat inactivation (80 °C for 5 min). The double-stranded cDNA was used as template for library construction using the Nextera XT library kit (Illumina, San Diego, CA, USA) with barcoded primers. The library was sequenced on a NextSeq 500 generating 2× 150 bp paired reads.

De novo assembly of the data yielded 10 million reads, 6.5M of which were assembled in a 5914 nucleotide-long genome sequence, which was identified as a macula-like virus (Genbank accession number MW959137). It was most closely related to the three published sequences for Hubei macula-like virus 3 (HMLV3) (Genbank accession numbers KX883799.1, KX883800.1 and MT240795.1) with 77–79% nucleotide identity over 64–93% of the query cover (*E*-values 0.0) using the blastn algorithm on NCBI BLAST [15,16]. HMLV3 was first identified from two spider samples as part of a large-scale metagenomics study in arthropods collected in China in 2016 [18]. Another strain was subsequently detected in soybean thrips collected in 2018 in the United States of America [19]. Another putative virus sequence shared a high identity with our sequence (72.35% identity over 90% of the sequence, *E*-value 0.0): Pyongtaek Culex Macula-like virus [20]. This sequence was discovered by metagenomics from *Cx. bitaeniorhynchus* (Giles, 1901) mosquitoes collected in South Korea in 2018.

The genus *Maculavirus* is part of the *Tymoviridae* family and the *Tymovirales* order and officially includes a single species recognized by the International Committee on Taxonomy of Viruses: Grapevine fleck virus [21]. The genome structure corresponds to macula viruses and macula-like viruses, with two main open reading frames (ORF). The first ORF (between nucleotide positions 20 and 4813) encodes a polyprotein, which contains methyltransferase, peptidase, helicase and RNA-dependent RNA polymerase domains, while the second ORF (between nucleotide positions 4887 and 5540) encodes for the coat protein [21].

We performed MUSCLE alignments of the available sequences for the three published strains of HMLV3 and Pyongtaek Culex Macula-like virus with our Australian sequence using Geneious Prime (Biomatters, Auckland, New Zealand). The overall nucleotide sequence alignment showed that our strain of HMLV3 shared 77% identity with the three published HMLV3 strains and 69% identity with Pyongtaek Culex Macula-like virus (see Table 4). Over the coat protein ORF, the Australian HMLV3 sequence shared over 88% identity with the published HMLV3 amino acid sequences and 77% identity with Pyongtaek Culex Macula-like virus. This showed that we had isolated a new strain of this virus, rather than a novel virus species, since the criteria demarcating species in the *Maculavirus* genus were determined by Martelli et al. as overall sequence identity under 70% and capsid protein sequence identity under 85% [21]. These data also confirm that Pyongtaek Culex Macula-like virus is a separate species, as its identity percentages match that description.

We designed a specific primer pair to screen our remaining 18 unknown virus-positive samples for this HMLV3 strain (forward: CCATGCAGAGCACTAGGATGC; reverse: CCACTAAGGATGCGAAGACC). The primer pair amplifies a 486 base pair amplicon in the 5′ region of the genome and was used with the SuperScript III One-Step RT-PCR System with Platinum Taq DNA Polymerase (Invitrogen, Thermo Fisher Scientific, Waltham, MA, USA) with a melting temperature of 50 °C and a 30-second extension time. One of the tested samples (DS25, a pool of 50 *An. bancroftii*) was identified as a second isolate of our HMLV3 strain, bringing the prevalence of this virus in our samples to 1.01% (2/198 pools) (see Table 2). Amplicon Sanger sequencing revealed that this isolate had seven nucleotide differences with the prototype strain over the 416 nucleotide-long amplicon (98.3% identity), which translated into a single amino acid difference (99.3% identity) (Genbank accession number MW959138). The close genetic relationship between isolates from different arthropods (two mosquito genera, spiders and thrips), and the fact that traditionally, maculaviruses replicate in plants, support the hypothesis that these viruses could be following a horizontal transmission cycle between plants and arthropods. This is also the working hypothesis for a number of other insect virus families, including viruses in the *Tymoviridae* family [14,22,23,24].

There are still 17 samples that remain MAVRIC positive and unidentified. These may contain new virus species within the families tested here, or novel viruses from insect virus families that have gone undetected in Australian mosquitoes so far. High throughput sequencing of RNA derived from these samples could help identify these unknown replicating viruses.

## 2. Conclusions

We have shown that human disease-centered mosquito surveillance can yield data on the mosquito virome, when using the optimized MAVRIC detection system [9]. We were able to show that a number of mosquito samples contained replicating viruses and identify three insect viruses from three distinct virus classes in these pools. We demonstrated the persistence of the insect-specific flavivirus Palm Creek virus in *Cq. xanthogaster* mosquitoes from the Darwin area over almost a decade, with limited genetic drift. We also detected a novel Hubei macula-like virus 3 strain in samples from two mosquito genera, suggesting the virus, originally detected in spiders and soybean thrips, might be involved in a horizontal transmission cycle between arthropods and plants. Overall, these data contribute to our general knowledge of the mosquito virome and insect viruses.

## Figures and Tables

**Table 1:** List and proportion of mosquito species of 4381 mosquitoes collected on two crocodile farms around Darwin between March and April 2018.

Species		Number of Collected Mosquitoes			Percentage of Collected Mosquitoes
Name	Author, Year	Farm 1	Farm 2	Total	Total
*Culex annulirostris*	Skuse, 1889	1261	1462	2723	62.15
*Mansonia uniformis*	Theobald, 1901	34	481	515	11.76
*Anopheles bancroftii*	Giles, 1902	5	305	310	7.08
*Culex palpalis*	Taylor, 1912	0	279	279	6.37
*Culex pullus*	Theobald, 1905	190	6	196	4.47
*Coquilletidia xanthogaster*	Edwards, 1924	44	44	88	2.01
*Culex quinquefasciatus*	Say, 1823	87	0	87	1.99
*Culex gelidus*	Theobald, 1901	58	0	58	1.32
*Culex bitaeniorhynchus*	Giles, 1901	1	48	49	1.12
*Culex vishnui* group	-	14	0	14	0.32
*Uranotaenia albescens*	Taylor, 1914	12	0	12	0.27
*Aedes alboscutellatus*	Theobald, 1907	6	0	6	0.14
*Aedes kochi*	Donitz, 1901	5	0	5	0.11
*Culex sitiens*	Wiedermann, 1828	0	4	4	0.09
*Aedes notoscriptus*	Skuse, 1889	1	2	3	0.07
*Culex (Lophoceramyia) * species	-	0	2	2	0.05
*Aedeomyia catasticta*	Knab, 1909	1	0	1	0.02
*Anopheles annulipes sensu lato*	Walker, 1856	0	1	1	0.02
*Tripteroides magnesianus*	Edwards, 1924	0	1	1	0.02
*Uranotaenia pygmaea*	Theobald, 1901	0	1	1	0.02
*Verrallina funerea*	Theobald, 1903	1	0	1	0.02
Damaged species	-	0	25	25	0.57
TOTAL		1720	2661	4381	100

**Table 2:** List of MAVRIC-positive samples with identified viruses out of the 198 mosquito pools.

Virus Identified	Mosquito Species	Mosquitoes per Pool	Pool Number	Date	Farm
AMNV-1	*Cx. quinquefasciatus*	17	D138	29.03.18	1
AMNV-1	*Cx. pullus*	47	D192	18.04.18	1
AMNV-1	*Cx. quinquefasciatus*	37	D194	18.04.18	1
PCV	*Cq. xanthogaster*	11	DS26	15.03.18	2
PCV	*Cq. xanthogaster*	1	D149	29.03.18	1
PCV	*Cq. xanthogaster*	11	D190	18.04.18	1
PCV	*Cq. xanthogaster*	5	D209	27.04.18	1
PCV	*Cq. xanthogaster*	5	D221	27.04.18	1
PCV	*Cq. xanthogaster*	13	D232	27.04.18	2
HMLV3	*Cx. annulirostris*	8	DS16	15.03.18	2
HMLV3	*An. bancroftii*	50	DS25	15.03.18	2

HMLV3: Hubei macula-like virus 3; PCV: Palm Creek virus; AMNV-1: alphamesonivirus-1 Nam Dinh virus. *Cx.: Culex; Cq.: Coquillettidia; An.: Anopheles.*

**Table 3:** Percentage identity between PCV isolates in the NS5 region amplified by FU2/cFD3 primers. Top right half displays amino acid identities (252 amino acid-long alignment), bottom left half displays nucleotide identities (737 nucleotide-long alignment). The names of isolate from this study start with a D.

	D190	56	D209	DS26	K71061	D232	D221	D149
**D190**		99.6	99.6	99.2	99.2	99.6	99.2	99.2
**56**	98.8		100	99.6	99.6	100	99.6	99.6
**D209**	98.9	99.3		99.6	99.6	100	99.6	99.6
**DS26**	95.6	96.3	95.6		100	99.6	100	100
**K71061**	95.2	95.9	95.2	99.3		99.6	100	100
**D232**	96.6	97.0	96.6	98.2	98.0		99.6	99.6
**D221**	96.0	96.4	96.0	98.5	98.2	98.3		100
**D149**	95.8	96.2	95.8	98.5	98.4	98.5	99.7	

Genbank accession numbers: PCV 56 KC505248.1, PCV K71061 KT192550.1, PCV DS26 MW959131, PCV D149 MW959132, PCV D190 MW959133, PCV D209 MW959134, PCV D221 MW959135, PCV D232 MW959136.

**Table 4:** Percentage identity between the Australian HMLV3 sequence and other HMLV3 strains and Pyongtaek Culex Macula-like virus. Top right half displays amino acid identities over the coat protein (217 amino acid-long alignment); bottom left half displays nucleotide identities over the whole available sequence (6061 nucleotide-long alignment).

	PCMLV	HMLV3 Spider 1	HMLV3 Spider 2	HMLV3 Thrip	HMLV3 Darwin
**PCMLV**		80.6	77.4	N/A	77.0
**HMLV3 spider 1**	70.5		88.5	N/A	88.5
**HMLV3 spider 2**	69.0	77.1		N/A	95.0
**HMLV3 thrip**	69.5	94.3	76.4		N/A
**HMLV3 Darwin**	69.2	77.2	76.8	77.0	

HMLV3: Hubei macula-like virus 3; PCMLV: Pyongtaek Culex Macula-like virus. Genbank accession numbers: PCMLV: MT568534; HMLV3 spider 1: KX883799; HMLV3 spider 2: KX883800; HMLV3 thrip: MT240795; HMLV3 Darwin: MW959137. N/A: not applicable, this sequence does not include a functional coat protein ORF.

## Data Availability

Genbank accession numbers for new sequences: MW959131, MW959132, MW959133, MW959134, MW959135, MW959136, MW959137, MW959138.

## References

[1] Whelan P.I. (2007). Mosquito Vector Control in the Northern Territory. North. Territ. Dis. Control Bull..

[2] Medical Entomology Centre for Disease Control Department of Health Northern Territory Government (2015). Medical Entomology Annual Report 2013/14.

[3] Isberg S.R., Moran J.L., De Araujo R., Elliott N., Davis S.S., Melville L. (2019). First Evidence of Kunjin Strain of West Nile Virus Associated with Saltwater Crocodile (*Crocodylus porosus*) Skin Lesions. Aust. Vet. J..

[4] Habarugira G., Moran J., Colmant A.M.G., Davis S.S., O’Brien C.A., Hall-Mendelin S., McMahon J., Hewitson G., Nair N., Barcelon J. (2020). Mosquito-Independent Transmission of West Nile Virus in Farmed Saltwater Crocodiles (*Crocodylus porosus*). Viruses.

[5] Kurucz N., McMahon J.L., Warchot A., Hewitson G., Barcelon J., Moore F., Moran J., Harrison J.J., Colmant A.M.G., Staunton K.M. (2022). Nucleic Acid Preservation Card Surveillance Is Effective for Monitoring Arbovirus Transmission on Crocodile Farms and Provides a One Health Benefit to Northern Australia. Viruses.

[6] Gentry M.K., Henchal E.A., McCown J.M., Brandt W.E., Dalrymple J.M. (1982). Identification of Distinct Antigenic Determinants on Dengue-2 Virus Using Monoclonal Antibodies. Am. J. Trop. Med. Hyg..

[7] Clark D.C., Lobigs M., Lee E., Howard M.J., Clark K., Blitvich B.J., Hall R.A. (2007). In Situ Reactions of Monoclonal Antibodies with a Viable Mutant of Murray Valley Encephalitis Virus Reveal an Absence of Dimeric NS1 Protein. J. Gen. Virol..

[8] O’Brien C.A., Hobson-Peters J., Yam A.W.Y., Colmant A.M.G., McLean B.J., Prow N.A., Watterson D., Hall-Mendelin S., Warrilow D., Ng M.-L. (2015). Viral RNA Intermediates as Targets for Detection and Discovery of Novel and Emerging Mosquito-Borne Viruses. PLoS Negl. Trop. Dis..

[9] O’Brien C.A., Harrison J.J., Colmant A.M.G., Traves R.J., Paramitha D., Hall-Mendelin S., Bielefeldt-Ohmann H., Vet L.J., Piyasena T.B.H., Newton N.D. (2021). Improved Detection of Flaviviruses in Australian Mosquito Populations via Replicative Intermediates. J. Gen. Virol..

[10] Prow N.A., Mah M.G., Deerain J.M., Warrilow D., Colmant A.M.G., O’Brien C.A., Harrison J.J., McLean B.J., Hewlett E.K., Piyasena T.B.H. (2018). New Genotypes of Liao Ning Virus (LNV) in Australia Exhibit an Insect-Specific Phenotype. J. Gen. Virol..

[11] Colmant A.M.G., O’Brien C.A., Newton N.D., Watterson D., Hardy J., Coulibaly F., Bielefeldt-Ohmann H., Warrilow D., Huang B., Paramitha D. (2020). Novel Monoclonal Antibodies against Australian Strains of Negeviruses and Insights into Virus Structure, Replication and Host -Restriction. J. Gen. Virol..

[12] O’Brien C.A., McLean B.J., Colmant A.M.G., Harrison J.J., Hall-Mendelin S., van den Hurk A.F., Johansen C.A., Watterson D., Bielefeldt-Ohmann H., Newton N.D. (2017). Discovery and Characterisation of Castlerea Virus, a New Species of Negevirus Isolated in Australia. Evol. Bioinform. Online.

[13] O’Brien C.A., Pegg C.L., Nouwens A.S., Bielefeldt-Ohmann H., Huang B., Warrilow D., Harrison J.J., Haniotis J., Schulz B.L., Paramitha D. (2020). A Unique Relative of Rotifer Birnavirus Isolated from Australian Mosquitoes. Viruses.

[14] Newton N.D., Colmant A.M.G., O’Brien C.A., Ledger E., Paramitha D., Bielefeldt-Ohmann H., Watterson D., McLean B.J., Hall-Mendelin S., Warrilow D. (2020). Genetic, Morphological and Antigenic Relationships between Mesonivirus Isolates from Australian Mosquitoes and Evidence for Their Horizontal Transmission. Viruses.

[15] Kuno G., Chang G.J., Tsuchiya K.R., Karabatsos N., Cropp C.B. (1998). Phylogeny of the Genus *Flavivirus*. J. Virol..

[16] Hobson-Peters J., Yam A.W.Y., Lu J.W.F., Setoh Y.X., May F.J., Kurucz N., Walsh S., Prow N.A., Davis S.S., Weir R. (2013). A New Insect-Specific Flavivirus from Northern Australia Suppresses Replication of West Nile Virus and Murray Valley Encephalitis Virus in Co-Infected Mosquito Cells. PLoS ONE.

[17] McLean B.J., Hobson-Peters J., Webb C.E., Watterson D., Prow N.A., Nguyen H.D., Hall-Mendelin S., Warrilow D., Johansen C.A., Jansen C.C. (2015). A Novel Insect-Specific Flavivirus Replicates Only in *Aedes*-Derived Cells and Persists at High Prevalence in Wild *Aedes vigilax* Populations in Sydney, Australia. Virology.

[18] Shi M., Lin X.-D., Tian J.-H., Chen L.-J., Chen X., Li C.-X., Qin X.-C., Li J., Cao J.-P., Eden J.-S. (2016). Redefining the Invertebrate RNA Virosphere. Nature.

[19] Thekke-Veetil T., Lagos-Kutz D., McCoppin N.K., Hartman G.L., Ju H.-K., Lim H.-S., Domier L.L. (2020). Soybean Thrips (*Thysanoptera*: *Thripidae*) Harbor Highly Diverse Populations of Arthropod, Fungal and Plant Viruses. Viruses.

[20] Sanborn M.A., Wuertz K.M., Kim H.-C., Yang Y., Li T., Pollett S.D., Jarman R.G., Berry I.M., Klein T.A., Hang J. (2021). Metagenomic Analysis Reveals *Culex* Mosquito Virome Diversity and Japanese Encephalitis Genotype V in the Republic of Korea. Mol. Ecol..

[21] Martelli G.P., Sabanadzovic S., Abou Ghanem-Sabanadzovic N., Saldarelli P. (2002). *Maculavirus*, a New Genus of Plant Viruses. Arch. Virol..

[22] Martelli G.P., Sabanadzovic S., Abou-Ghanem Sabanadzovic N., Edwards M.C., Dreher T. (2002). The Family *Tymoviridae*. Arch. Virol..

[23] Agboli E., Leggewie M., Altinli M., Schnettler E. (2019). Mosquito-Specific Viruses—Transmission and Interaction. Viruses.

[24] Colmant A.M.G., Furlong M.J., Etebari K. (2022). Discovery of a Novel Jingmenvirus in Australian Sugarcane Soldier Fly (*Inopus flavus*) Larvae. Viruses.

